# Hydrogen Sulfide Oxidation: Adaptive Changes in Mitochondria of SW480 Colorectal Cancer Cells upon Exposure to Hypoxia

**DOI:** 10.1155/2019/8102936

**Published:** 2019-01-29

**Authors:** Francesca Malagrinò, Karim Zuhra, Ludovica Mascolo, Daniela Mastronicola, João B. Vicente, Elena Forte, Alessandro Giuffrè

**Affiliations:** ^1^Department of Biochemical Sciences, Sapienza University of Rome, Rome, Italy; ^2^CNR Institute of Molecular Biology and Pathology, Rome, Italy; ^3^Instituto de Tecnologia Química e Biológica António Xavier, Universidade Nova de Lisboa, Oeiras, Portugal

## Abstract

Hydrogen sulfide (H_2_S), a known inhibitor of cytochrome *c* oxidase (CcOX), plays a key signaling role in human (patho)physiology. H_2_S is synthesized endogenously and mainly metabolized by a mitochondrial sulfide-oxidizing pathway including sulfide:quinone oxidoreductase (SQR), whereby H_2_S-derived electrons are injected into the respiratory chain stimulating O_2_ consumption and ATP synthesis. Under hypoxic conditions, H_2_S has higher stability and is synthesized at higher levels with protective effects for the cell. Herein, working on SW480 colon cancer cells, we evaluated the effect of hypoxia on the ability of cells to metabolize H_2_S. The sulfide-oxidizing activity was assessed by high-resolution respirometry, measuring the stimulatory effect of sulfide on rotenone-inhibited cell respiration in the absence or presence of antimycin A. Compared to cells grown under normoxic conditions (air O_2_), cells exposed for 24 h to hypoxia (1% O_2_) displayed a 1.3-fold reduction in maximal sulfide-oxidizing activity and 2.7-fold lower basal O_2_ respiration. Based on citrate synthase activity assays, mitochondria of hypoxia-treated cells were 1.8-fold less abundant and displayed 1.4-fold higher maximal sulfide-oxidizing activity and 2.6-fold enrichment in SQR as evaluated by immunoblotting. We speculate that under hypoxic conditions mitochondria undergo these adaptive changes to protect cell respiration from H_2_S poisoning.

## 1. Introduction

Hydrogen sulfide (H_2_S) has been increasingly recognized as a key signaling molecule in human (patho)physiology. While being able to regulate cell redox homeostasis and other crucial physiological functions at low (nM) concentrations [1–4], at higher (*μ*M) levels, H_2_S exerts toxicity both inhibiting O_2_ consumption by cytochrome *c* oxidase (CcOX) in the mitochondrial electron transport chain [5] and impairing O_2_ transport/storage through covalent modification of the heme porphyrin ring in globins (reviewed in [6]). It is therefore crucial that cells tightly control H_2_S bioavailability to prevent toxicity.

In humans, at least three enzymes are directly involved in H_2_S synthesis (reviewed in [1, 7, 8]): cystathionine *β*-synthase (CBS) and cystathionine *γ*-lyase (CSE), belonging to the transulfuration pathway, and 3-mercaptopyruvate sulfurtransferase (MST). Of these, CBS is inhibited with relatively high affinity by nitric oxide (NO) and carbon monoxide (CO), particularly in the presence of the allosteric stimulator S-adenosyl-L-methionine [9–13]. H_2_S breakdown is instead mostly accomplished by a mitochondrial enzymatic pathway that couples the oxidation of H_2_S into thiosulfate (S_2_O_3_^2-^) and sulfate (SO_4_^2-^) to ATP synthesis [14]. The first step of sulfide breakdown is catalyzed by the membrane-associated sulfide:quinone oxidoreductase (SQR). This flavoprotein transfers electrons from H_2_S to coenzyme Q in the mitochondrial electron transfer chain, thus making H_2_S the first inorganic substrate that is able to sustain mitochondrial respiration [15]. Concomitantly, SQR transfers the H_2_S sulfur atom to an acceptor, leading to the formation of glutathione persulfide (GSSH) [16, 17] or, less likely, S_2_O_3_^2-^ [18, 19]. Differences in the SQR substrate specificity were recently reported comparing the soluble with the nanodisc-incorporated enzyme [20]. Three additional enzymes, persulfide dioxygenase (ETHE1), thiosulfate sulfurtransferase, and sulfite oxidase, cooperate with SQR in the mitochondrial sulfide oxidation pathway, to oxidize H_2_S into SO_4_^2-^ and S_2_O_3_^2-^. To process 1 H_2_S molecule, mitochondria overall consume ~0.75 O_2_ molecules (0.25 by CcOX plus 0.5 by ETHE1, [21]). Besides being metabolized through the mitochondrial sulfide-oxidizing pathway, H_2_S can be oxidized by several metalloproteins such as globins, heme-based sensors of diatomic gaseous molecules, catalase, and peroxidases (see [8] and references therein) or be catabolized by the cytosolic thiol methyltransferase [22].

*In vivo*, H_2_S can therefore exert a dual effect on cell bioenergetics, at lower concentrations stimulating *via* SQR mitochondrial respiration and thus ATP synthesis or causing a reversible inhibition of CcOX at higher concentrations (reviewed in [23–26]). Notably, the sulfide-oxidizing activity varies considerably between different cell types and tissues, spanning from undetectable, as e.g., in neuroblastoma cells, to high, as observed in colonocytes [15, 21, 27]. The high H_2_S-detoxifying ability of colonocytes is perhaps not surprising as these cells are physiologically exposed to the fairly high H_2_S levels produced by the gut microbiota (reviewed in [28]).

Among other diseases, cancer has been increasingly associated with alterations of H_2_S metabolism [29–31]. In particular, CBS has been shown to be overexpressed in cell lines and samples of colorectal cancer [32] and other cancer types [33–36]. In colorectal cancer cell lines, CBS-derived H_2_S was proposed to promote cell proliferation and angiogenesis and to sustain cellular bioenergetics by stimulating both oxidative phosphorylation and glycolytic ATP synthesis. The enzyme is therefore currently recognized as a drug target [29, 31, 37]. CSE and CSE-derived H_2_S have been recognized as key elements in melanoma progression [38]. All three H_2_S-synthesizing enzymes have been posited to contribute to the correlation between increased H_2_S production and tumor stage and grade in bladder urothelial cell carcinoma [39]. Moreover, Szczesny et al. [36] observed higher expression levels of all three H_2_S-generating enzymes and increased H_2_S-producing activity in lung adenocarcinoma samples as compared to the adjacent normal lung tissue. A link between H_2_S production and mitochondrial DNA repair was proposed, and the inhibition of CBS and CSE by aminooxyacetic acid or siRNA-mediated depletion of CBS, CSE, or MST in the lung adenocarcinoma A549 cell line resulted in compromised integrity of mitochondrial DNA. Irrespectively of the downstream mechanisms linking increased H_2_S levels and cell proliferation and/or tumor progression, it remains to be established how cancer cells circumvent the potentially toxic effects of increased H_2_S.

Hypoxia is a common factor in the microenvironment of solid tumors that has been recognized to be associated to drug resistance and promotion of cancer progression, metastasization, and angiogenesis (see [40] for a review). The effect of hypoxia on cancer metabolism has been extensively investigated (reviewed in [41–43]). Among other changes, hypoxic cells undergo a reduction in mitochondrial mass, resulting from reduced biogenesis of this organelle and enhanced mitophagy [44–46]. Because mitochondria are the main site of sulfide oxidation, in the absence of compensatory mechanisms, hypoxic cells are expected to display a reduced ability to detoxify sulfide. The intricate interplay between H_2_S and O_2_ has been extensively investigated (reviewed in [47, 48]). As O_2_ facilitates both the chemical and enzymatic oxidative decomposition of H_2_S into persulfides and polysulfides, at low O_2_ tension a higher stability of H_2_S is expected. Furthermore, hypoxic/ischemic conditions have been reported to enhance H_2_S synthesis, through upregulation or stimulation of the sulfide-synthesizing enzymes [49, 50], accumulation of CBS in mitochondria, likely augmenting the H_2_S mitochondrial levels [51], and release of CO-mediated inhibition of CBS and CSE [52, 53]. Hypoxia is thus expected to increase H_2_S bioavailability, a condition that can have opposite physiological consequences. Indeed, while H_2_S has been shown to be protective against ischemic injuries [54, 55], the enhanced biosynthesis and chemical stability of H_2_S, combined with the reduced content in mitochondria (the main sites of sulfide disposal), may increase the risk of H_2_S toxicity in hypoxic cells.

This information prompted us to investigate in the present study the effect of hypoxia on the mitochondrial sulfide-oxidizing activity and SQR expression in colorectal cancer cells.

## 2. Materials and Methods

### 2.1. Materials

The human colon cancer cell line SW480 was purchased from the American Type Culture Collection (ATCC no. CCL228™). Sodium sulfide nonahydrate (Na_2_S·9H_2_O, 431648), 5,5′-dithiobis-(2-nitrobenzoic acid) (DTNB), acetyl coenzyme A, oxaloacetate, CelLytic™ MT cell lysis reagent, protease inhibitor cocktail (P8340), and rabbit polyclonal antibody against human SQR (HPA017079) were purchased from Sigma. The bicinchoninic acid assay (BCA) kit was from Thermo Fisher Scientific. Cell culture media and antibiotics were from Sigma, EuroClone, or Gibco. Mini-PROTEAN TGX Stain-Free Precast Gels, the Clarity Western ECL Substrate, and the Laemmli protein sample buffer were purchased from Bio-Rad. Bovine serum albumin was from AppliChem.

### 2.2. Preparation of Sulfide Stock Solutions

Stock solutions of Na_2_S were prepared by quickly washing the surface of a crystal of sodium sulfide nonahydrate with degassed ultrapure (Milli-Q®) water and then dissolving it in degassed Milli-Q water under N_2_ atmosphere, as reported in [56]. The concentration of Na_2_S in solution was measured spectrophotometrically using 5,5′-dithiobis-(2-nitrobenzoic acid) (DTNB) according to Nashef et al. [57] in a Cary 60 UV-VIS spectrophotometer. The concentration of Na_2_S was then adjusted to 3-5 mM by dilution with degassed ultrapure (Milli-Q®) water in a gas-tight glass syringe.

### 2.3. Cell Culture

The human colon cancer cell line SW480 was maintained in Dulbecco's Modified Eagle Medium (DMEM) containing 4.5 g·L^−1^ glucose, supplemented with 2 mM l-glutamine, 10% (*v*/*v*) heat-inactivated fetal bovine serum (FBS), 100 U·mL^−1^ penicillin, and 100 *μ*g·mL^−1^ streptomycin. Cells at 37°C and 5% CO_2_ in 25 cm^2^ or 75 cm^2^ flasks were grown under normoxic conditions (air O_2_) or incubated for 24 h under hypoxic conditions (1% O_2_) in a Galaxy 14 S incubator (Eppendorf) designed to maintain cell cultures at controlled O_2_ tension. After trypsinization, the cells were washed in the culture medium, counted using the trypan blue dye exclusion test, centrifuged at 1000 ×g for 5 min, and resuspended in fresh medium at a final density of 8 × 10^6^ cells·mL^−1^. Trypan blue-positive cells were always less than 5%. Cells grown under air conditions or exposed to hypoxia are, respectively, referred to as “normoxic” and “hypoxia-treated” cells.

### 2.4. Measurements of the Mitochondrial Sulfide-Oxidizing Activity

The mitochondrial sulfide-oxidizing activity of tested cells was evaluated as described in [25], by measuring the stimulatory effect of sulfide on cellular O_2_ consumption. Measurements were carried out at 37°C, using a high-resolution respirometer (Oxygraph-2k, Oroboros Instruments, Innsbruck, Austria), equipped with two 1.5 mL chambers and a micropump (TIP-2k) allowing for steady injections of relatively small amounts of sulfide into the chambers. According to Abou-Hamdan et al. [25], in these assays, sulfide is injected into a cell suspension at increasing flux (determined by the pump rate) and the mitochondrial sulfide-detoxifying activity is evaluated from the observed stimulation of cellular O_2_ consumption. Indeed, upon increasing the rate of sulfide injection, the concentration of sulfide in solution and, in turn, the sulfide-sustained cellular O_2_ consumption increase until the concentration of injected sulfide becomes inhibitory for CcOX. In colorectal cancer cells, SQR-mediated sulfide detoxification was shown to promote both forward electron transfer to O_2_ via quinol:cytochrome *c* reductase (complex III)/cytochrome *c*/CcOX and reverse electron transfer through complex I [21]. Therefore, measurements were herein carried out in the presence of rotenone, a known inhibitor of complex I, to prevent electrons derived from SQR-mediated sulfide oxidation to be partially diverted from O_2_ reduction with consequent underestimation of the mitochondrial sulfide-oxidizing activity. Herein, the assays were typically conducted in FBS-supplemented cell medium under stirring as follows. A suspension of four million cells was added into the respirometer chamber, and the basal respiration was measured for ~10 min. Afterwards, following the addition of 5 *μ*M rotenone resulting in O_2_ consumption inhibition, a solution of 3-5 mM sulfide was injected for time intervals of 180 s at increasing rates (10 nL·s^−1^, 20 nL·s^−1^, 40 nL·s^−1^, 80 nL·s^−1^, and 160 nL·s^−1^) and the effect on O_2_ consumption was measured. Control experiments were carried out in the presence of both rotenone (5 *μ*M) and antimycin A (5 *μ*M), an inhibitor of complex III. The latter assays allowed us to evaluate the effect of sulfide on extramitochondrial and nonenzymatic O_2_ consumption and thus obtain by subtraction (from the experiments performed in the absence of antimycin A) the genuine mitochondrial O_2_ consumption activity due to sulfide oxidation and from it an estimate of the H_2_S-oxidizing activity, considering that ~1.33 molecules of H_2_S per O_2_ molecule are reportedly consumed by the mitochondrial sulfide-oxidizing pathway [21].

### 2.5. Evaluation of Mitochondrial Content by the Citrate Synthase Assay

Cells were harvested and lysed using the CelLytic™ MT cell lysis reagent and protease inhibitor cocktail from Sigma according to the manufacturer's instructions. Cell extracts were assayed spectrophotometrically for citrate synthase in 100 mM Tris-HCl, 0.3 mM acetyl-CoA, 0.1 mM DTNB and 0.1 mM oxaloacetate, as described in [58].

### 2.6. Immunoblotting Assays

Cells were harvested and lysed as described in the previous section, and after total protein content determination by the bicinchoninic acid method, proteins (20 *μ*g per lane) were separated by SDS-PAGE using Mini-PROTEAN TGX Stain-Free Precast Gels (Bio-Rad). The formulation of these gels includes trihalo compounds which lead to UV fluorescence emission upon reaction with proteins [59], allowing estimation of the total protein load in a gel lane, using a ChemiDoc MP imaging system (Bio-Rad) without resorting to staining procedures or housekeeping proteins for normalization purposes. Proteins commonly used as housekeepers, such as glyceraldehyde 3-phosphate dehydrogenase and *β*-actin, indeed are known to change their expression levels under hypoxia [60, 61]. Afterwards, the proteins separated by SDS-PAGE were transferred onto a polyvinylidene difluoride membrane using a Trans-Blot SD Semi-Dry Electrophoretic Transfer Cell (from Bio-Rad) at 180 mA for 30 min. The membrane was blocked with PBS-T (phosphate-buffered saline with 0.1% Tween 20 (*v*/*v*)) containing 3% bovine serum albumin (BSA, *w*/*v*) and then incubated overnight at 4°C with the antibody against human SQR (1 : 150, in PBS-T with 3% BSA (*w*/*v*)). After three washing steps with PBS-T (15 min), the membrane was incubated with horseradish peroxidase-conjugated secondary antibody (1 : 5000, in PBS-T with 3% BSA (*w*/*v*)), followed by three washing steps with PBS-T (15 min) and detection by enhanced chemiluminescence (Clarity Western ECL Substrate, Bio-Rad). Finally, the blotted membrane was subjected to densitometric analysis using the Image Lab software (Bio-Rad), followed by the normalization of the target protein band intensity to the total protein load determined as described above.

### 2.7. Data Analysis

Oxygen consumption rates (OCR) were calculated using the software DatLab4 (Oroboros Instruments, Austria). Data are reported as mean ± standard error of the mean (SEM). Statistical significance (*P*) was estimated using Student's *t*-test in Microsoft Excel. ^∗^*P* ≤ 0.05, ^∗∗^*P* ≤ 0.01, and ^∗∗∗^*P* ≤ 0.001 were considered significant.

## 3. Results

**Figure 1:**
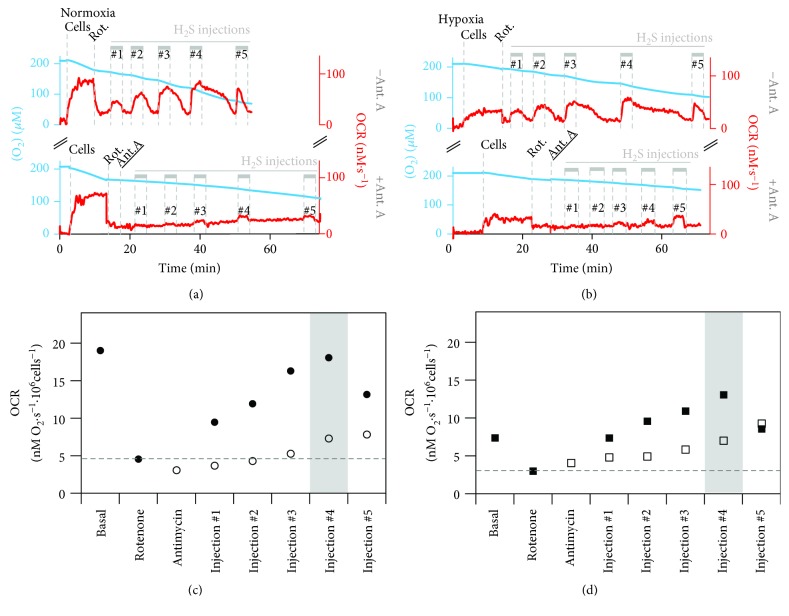
Stimulation of O_2_ consumption by sulfide. Representative oxygen consumption traces (blue) and corresponding O_2_ consumption rate (OCR, red traces) acquired with normoxic (a) or hypoxia-treated SW480 cells (b), following the addition of cells (4 × 10^6^), rotenone (Rot., 5 *μ*M) either alone (top traces) or plus antimycin A (Ant. A, 5 *μ*M, bottom traces), and subsequent injection of a sulfide solution (3-5 mM) at increasing rates (10 nL·s^−1^, 20 nL·s^−1^, 40 nL·s^−1^, 80 nL·s^−1^, and 160 nL·s^−1^, corresponding, respectively, to injections #1 to #5). (c, d) OCR values obtained from the oxygraphic traces, respectively shown in (a) and (b), measured at basal condition and upon sulfide injection at increasing rates after addition of rotenone alone (full symbols) or rotenone plus antimycin A (hollow symbols). Mitochondrial H_2_S consumption in normoxic cells was calculated by determining the OCR measured at the highest non-inhibitory H_2_S injection rate (highlighted with grey bar in (c)) and subtracting the OCR measured after the addition of rotenone (horizontal dashed line in (c)), yielding ΔOCR_(-Ant)_. Then, the ΔOCR at the corresponding sulfide injection in the antimycin A-containing measurement was calculated in the same manner, yielding ΔOCR_(+Ant)_. By calculating ΔOCR_(‐Ant)_ − ΔOCR_(+Ant)_, the genuine mitochondrial H_2_S-dependent OCR (OCR_mitH2S_) was determined. Finally, OCR_mitH2S_ was multiplied by 1.33 to account for the number of H_2_S molecules consumed per O_2_ molecule, yielding an estimated sulfide oxidizing activity of 12.7 nM H_2_S·s^−1^·10^6^ cells^−1^. Employing the same procedure for cells exposed to hypoxia (b, d), an activity of 9.5 nM H_2_S·s^−1^·10^6^ cells^−1^ was estimated.

Colorectal cancer SW480 cells were either grown under normoxic (air O_2_) conditions or exposed for 24 h to hypoxia (1% O_2_), and their sulfide-oxidizing activity was assayed by high-resolution respirometry, according to Abou-Hamdan et al. [25], as described in Materials and Methods. A representative oxygraphic trace acquired with untreated (“normoxic”) cells is shown in Figure 1(a). The trace shows that ~80% of oxygen consumption was blocked by the addition of the complex I inhibitor rotenone, added to prevent sulfide oxidation through reversal of complex I activity, as described in [21, 62]. Sulfide was then injected five times at increasing rates into the oxygraphic chamber via a micropump. The first four injections led to the stimulation of O_2_ consumption, pointing to a fully operative mitochondrial sulfide-oxidizing pathway in the tested cells (Figures 1(a) and 1(b)). The stimulation persisted for the entire duration (3 minutes) of sulfide injection, after which the O_2_ consumption rate (OCR) declined back to the value measured in the absence of sulfide. The decline took a few minutes, as if some sulfide persisted in solution, sustaining cell respiration even after the injection was stopped. The extent of O_2_ consumption stimulation by sulfide increased with the rate of sulfide injection (up to 80 nL·s^−1^, Figures 1(a) and 1(c)). However, upon further increasing the injection rate (to 160 nL·s^−1^), a decline in OCR was observed already before sulfide injection was stopped, likely due to CcOX inhibition by sulfide, as suggested previously [25].

For comparison, the measurements described above were carried out on the same cells after 24 h exposure to hypoxic conditions. A representative oxygraphic trace is shown in Figure 1(b). Hypoxia-treated cells displayed a lower basal respiratory activity compared to untreated cells (6.3 ± 0.5 nM O_2_·s^−1^ vs. 17.1 ± 1.1 nM O_2_·s^−1^ per million cells). Yet, as observed for normoxic cells, after rotenone addition a progressive stimulation of cell respiration was observed upon injecting sulfide at an increasing rate (Figures 1(b) and 1(d)), until the amount of injected sulfide exceeded the detoxifying activity of the cells, and CcOX inhibition occurred, leading to impairment of cell respiration (see last sulfide injection in Figure 1(b), top).

**Figure 2:**
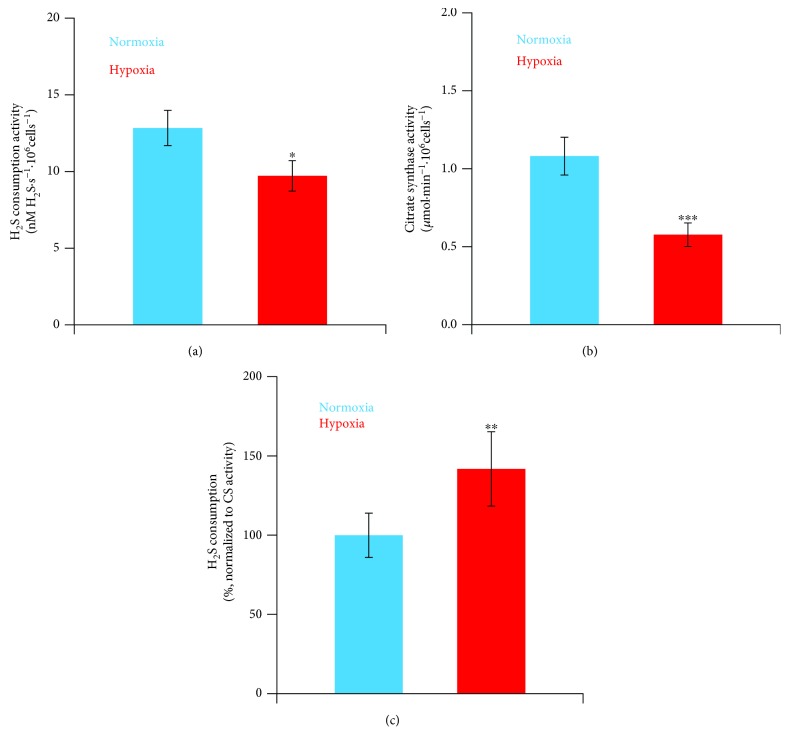
Effect of hypoxia on mitochondrial sulfide consumption. (a) Mean values of maximal estimated sulfide consumption activity (calculated as described in the legend of Figure 1(c)), measured in normoxic (*n* = 9, blue bar) and hypoxia-treated (*n* = 8, red bar) cells. (b) Citrate synthase activity in normoxic (*n* = 13, blue bar) and hypoxia-treated (*n* = 10, red bar) cell lysates. (c) Maximal sulfide consumption activity normalized to the citrate synthase activity, as measured in normoxic (blue bar) and hypoxia-treated (red bar) cells. ^∗^*P* ≤ 0.05; ^∗∗^*P* ≤ 0.01; ^∗∗∗^*P* ≤ 0.001.

**Figure 3:**
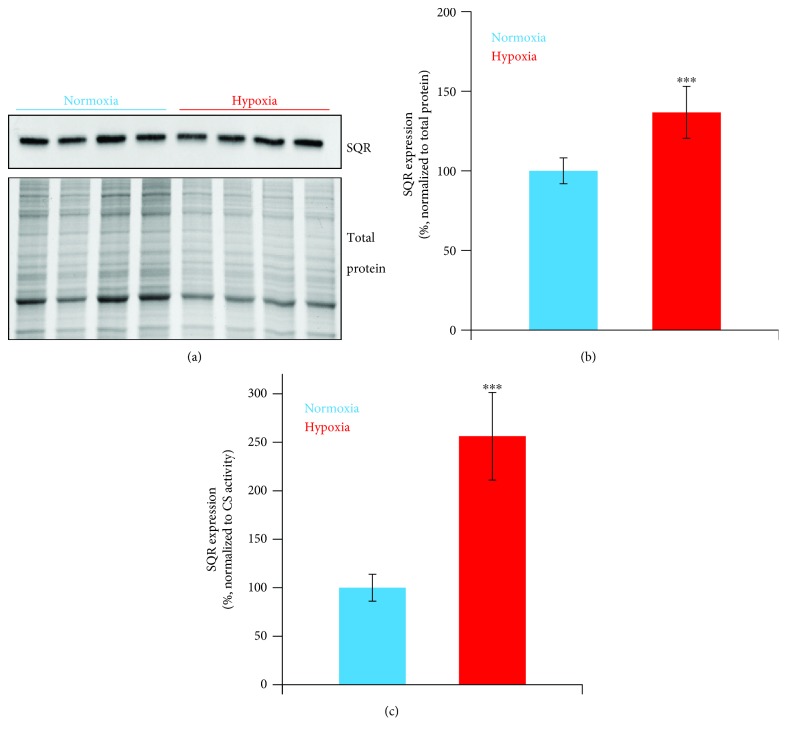
Effect of hypoxia on SQR expression. Representative Western blot analyzing SQR expression in normoxic and hypoxia-exposed SW480 cells (a), with the corresponding total protein load quantitation by stain-free imaging technology (see Materials and Methods). SQR levels in normoxic (*n* = 4 in triplicate, blue bars) and hypoxia-treated cells (*n* = 4 in triplicate, red bars), as normalized to total protein (b) or citrate synthase activity (c). ^∗∗∗^*P* ≤ 0.001.

To evaluate the contribution of mitochondria to the observed sulfide-oxidizing activity, we used antimycin A, a known inhibitor of complex III that blocks quinol oxidation in the respiratory chain and thus prevents sulfide oxidation by mitochondria [25]. As shown in Figures 1(a) and 1(b) (bottom traces), in the presence of rotenone, antimycin A considerably prevented O_2_ consumption stimulation by sulfide in both normoxic and hypoxia-treated cells, proving that under the tested conditions sulfide oxidation occurs mostly at the mitochondrial level. The effect of sulfide on mitochondrial O_2_ consumption was quantitatively evaluated by subtracting the OCR values measured during sulfide injection in the presence of both rotenone and antimycin A from those measured at identical sulfide injection rates in the presence of rotenone only (see legend of Figure 1 for more details). According to this analysis, at the highest non-inhibitory (for CcOX) injection rate sulfide sustained a mitochondrial O_2_ consumption of 9.7 ± 1.2 nM O_2_·s^−1^ and 7.3 ± 0.8 nM O_2_·s^−1^ per million cells, in normoxic and hypoxia-treated cells, respectively. Considering that the mitochondrial sulfide-oxidizing pathway overall was reported to consume ~1.33 molecules of H_2_S per O_2_ molecule [21], a mitochondrial sulfide-oxidizing activity of 12.8 ± 1.5 and 9.7 ± 1.1 nM H_2_S·s^−1^ per million cells was estimated for normoxic and hypoxia-treated cells, respectively (Figure 2(a)). To evaluate the mitochondrial content in the tested cells, we carried out citrate synthase activity assays, a validated surrogate biomarker of mitochondrial content ([63] and references therein). Normoxic and hypoxia-treated cells displayed, respectively, a citrate synthase activity of 1.1 ± 0.1 *μ*mol·min^−1^·10^6^ cells^−1^ and 0.6 ± 0.1 *μ*mol·min^−1^·10^6^ cells^−1^ (Figure 2(b)), consistent with a reduction in the mitochondrial content upon exposure to hypoxia [44–46]. The measured citrate synthase activity was used to normalize the calculated mitochondrial sulfide-oxidizing activity, which proved to be in hypoxia-treated cells ~1.4-fold higher than in normoxic cells (Figure 2(c)). Finally, we have assayed by immunoblotting combined with “*stain-free*” imaging technology the SQR expression level in the tested cells (Figure 3(a)) and found that hypoxia-treated cells display 1.4-fold higher SQR protein levels than normoxic cells (Figure 3(b)). Considering that hypoxia-treated cells have a lower mitochondrial content (based on citrate synthase activity assays, Figure 2(b)), we estimate that the mitochondria of hypoxia-treated cells contain 2.6-fold more SQR than those of normoxic cells (Figure 3(c)).

## 4. Discussion

O_2_ and H_2_S are key molecules in living systems, able to control each other's availability, and regulate numerous processes in human (patho)physiology. As reviewed in [47], the interplay between H_2_S and O_2_ is intricate and based on several mechanisms: (i) direct reaction between the two, (ii) O_2_-dependent H_2_S breakdown through the mitochondrial sulfide-oxidizing pathway, (iii) H_2_S-mediated stimulation or inhibition of mitochondrial O_2_ consumption, (iv) O_2_-dependent regulation of expression and cellular relocalization of the H_2_S-synthesizing enzymes, and (v) O_2_-dependent control of CO-mediated inhibition of H_2_S production by CBS. H_2_S has indeed been recognized as an O_2_ sensor [64]. Despite this, to our knowledge no studies have been conducted yet to explore the effect of prolonged exposure to hypoxia on the cell ability to dispose of H_2_S, which represented the main objective of the present study.

Under hypoxic conditions, H_2_S plays a key protective role against ischemia/reperfusion damages [54, 55] through only partly understood molecular mechanisms including induction of antioxidant and vasorelaxation effects on microcirculation. Moreover, H_2_S appears to mediate the repair of damaged mitochondrial DNA [36], occurring in ischemia/reperfusion, and to protect from hypoxia-induced proteostasis disruption, as demonstrated in *Caenorhabditis elegans* [65]. In knockdown experiments with Hepa1-6 cells, H_2_S-mediated protection during O_2_ deprivation was found to require SQR [66], pointing to a key role of H_2_S catabolism in the cellular protective responses to hypoxia. Consistently, under hypoxic conditions, thiosulfate, a major product of H_2_S oxidation, has been shown to exert protective effects against ischemia/reperfusion damage [66–68] and also to generate H_2_S [69]. In this context, it is noteworthy that H_2_S is able to mimic hypoxia-induced responses such as vasodilation [70], neoangiogenesis [71], and expression of the hypoxia-inducible factor (HIF-1*α*, [72]), a master gene regulator promoting cell survival under hypoxic conditions shown to stimulate CBS expression in hypoxia [49]. The occurrence of H_2_S under hypoxic conditions is therefore likely part of a more general adaptive response adopted by the cells to ensure survival and protection from damages resulting from O_2_ deprivation (and possible reoxygenation).

In hypoxic cells, H_2_S bioavailability therefore needs to be finely regulated for this gaseous molecule to occur at physiologically protective yet non-poisonous levels. In this regard, it seems relevant to gain insight into the regulation of H_2_S production and breakdown at low O_2_ tensions. Previous studies focused on the H_2_S-synthesizing enzymes have shown that, under hypoxic conditions, H_2_S synthesis is enhanced [47] through multiple mechanisms [49–53] (see Introduction). In addition, H_2_S breakdown via both chemical and enzymatic reaction pathways is negatively affected by low O_2_ tensions. Evidence for a lower mitochondrial sulfide-oxidizing activity at lower O_2_ concentrations was initially provided in [73] working on immortalized cells derived from alveolar macrophages and, then, corroborated by Abou-Hamdan et al. in a more recent investigation on CHO cells [74].

**Figure 4:**
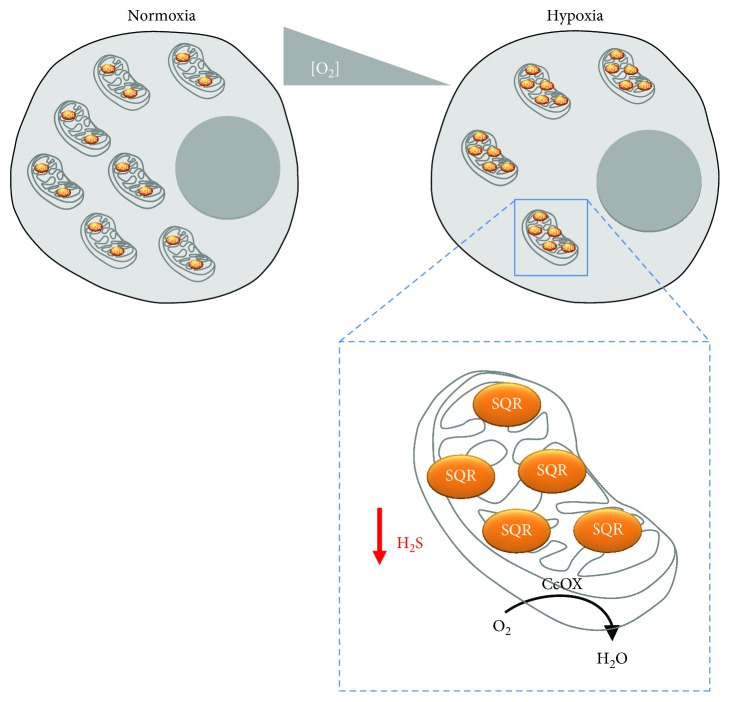
Adaptive changes occurring in mitochondria in response to hypoxia. Upon prolonged exposure to hypoxia, mitochondria become less abundant, but enriched in sulfide:quinone oxidoreductase (SQR). Consistently, their maximal sulfide-oxidizing activity increases, while overall decreasing in the cell. These changes are proposed to occur to prevent H_2_S inhibition of cytochrome *c* oxidase (CcOX) and thus protect cell respiration from H_2_S poisoning.

In the present study, using SW480 colorectal cancer cells as a model, we tested the effect of prolonged (24 h) exposure to 1% O_2_ on the cellular ability to dispose of sulfide at the mitochondrial level. Exposure to hypoxia leads to a notable (2.7-fold) reduction in basal respiration and to a marked (1.8-fold) decrease in the mitochondrial content (Figure 2(b)), as previously documented and suggested to result from enhanced mitophagic activity and reduced organelle biogenesis [44–46]. Hypoxia-treated cells also display a lower ability to dispose of H_2_S as compared to normoxic cells (Figure 2(a)). However, considering the above-mentioned decrease in mitochondrial content, the sulfide-detoxifying capacity of hypoxia-treated cells normalized to their minor mitochondrial content actually turned out to be 1.4-fold higher than that of untreated cells, pointing to an enhanced sulfide disposal capacity of mitochondria in hypoxia-treated cells. To gain further insight, we analyzed the SQR expression by immunoblotting, employing “*stain-free*” imaging technology for total protein quantitation and normalization purposes. Using this approach, we made the somewhat puzzling observation that hypoxia-treated cells, though displaying slightly reduced overall sulfide-oxidizing activity, have modestly (~1.4-fold) increased SQR levels. Interestingly, normalizing the SQR expression to the mitochondrial content revealed that, in line with their enhanced sulfide-oxidizing capacity, mitochondria of hypoxia-treated SW480 cells have ~2.6-fold higher levels of SQR than those of normoxic cells. Altogether, these results are intriguing in that they suggest that mitochondria in hypoxia-treated cells display lower mass but are enriched in SQR. The increased SQR levels could have a protective role in hypoxic cells preventing mitochondria to be poisoned by enhanced production of sulfide (Figure 4).

## 5. Conclusions

This is to our knowledge the first study in which the effect of prolonged cell exposure to hypoxia on the mitochondrial sulfide-oxidizing activity has been evaluated. The evidence collected here on SW480 colorectal cancer cells shows that hypoxia-treated cells metabolize sulfide with overall reduced maximal efficacy and have reduced mitochondrial content, but mitochondria are better equipped to dispose of H_2_S. Physiologically, this may represent a regulatory mechanism to ensure higher protective H_2_S levels, while protecting mitochondria from H_2_S toxicity.

## Data Availability

The data used to support the findings of this study are available from the corresponding authors upon request.

## Abbreviations

H_2_S: Hydrogen sulfide
SQR: Sulfide:quinone oxidoreductase
NO: Nitric oxide
CO: Carbon monoxide
CcOX: Cytochrome *c* oxidase
CBS: Cystathionine *β*-synthase
CSE: Cystathionine *γ*-lyase
MST: 3-Mercaptopyruvate sulfurtransferase
SO_4_^2-^: Sulfate
S_2_O_3_^2-^: Thiosulfate
DTNB: 5,5′-Dithiobis-(2-nitrobenzoic acid)
PBS-T: Phosphate-buffered saline with 0.1% Tween 20 (*v*/*v*)
OCR: Oxygen consumption rate.
